# Dynamics of an HIV model with cytotoxic T-lymphocyte memory

**DOI:** 10.1186/s13662-020-03035-8

**Published:** 2020-10-17

**Authors:** Chunhua Liu, Lei Kong

**Affiliations:** 1 School of Mathematics and Statistics, Yangtze Normal University, Fuling district, 408100 Chongqing city, P.R. China; 2 School of Mathematics and Statistics, Guizhou University of Finance and Economics, Guizhou, 550025 P.R. China

**Keywords:** HIV model, Routh–Hurwitz theorem, Central manifold, Bistability, Hopf bifurcation

## Abstract

We consider a four-dimensional HIV model that includes healthy cells, infected cells, primary cytotoxic T-lymphocyte response (CTLp), and secondary cytotoxic T-lymphocyte response (CTLe). The CTL memory generation depends on CD4^+^ T-cell help, and infection of CD4^+^ T cells results in impaired T-cell help. We show that the system has up to five equilibria. By the Routh–Hurwitz theorem and central manifold theorem we obtain some sufficient conditions for the local stability, globally stability of the equilibria, and the bifurcations. We still discover the bistability case where in the system there may coexist two stable equilibria or a stable equilibrium together with a stable limit cycle. Several numerical analyses are carried out to illustrate the validity of our theoretical results.

## Introduction

Human immunodeficiency virus (HIV) and acquired immunodeficiency syndrome (AIDS) have become global health problems; there were estimated 38.0 million people living with HIV at the end of 2019 [1]. HIV can be transmitted via the exchange of a variety of body fluids from infected people, such as blood, breast milk, or semen and vaginal secretions, but it is known that current antiretroviral drugs cannot enucleate HIV from the body. Mathematical models have been formulated for various epidemiological diseases like novel coronavirus (COVID-19) [2–4] or malaria [5]. Models of HIV as a chronic infectious disease have been investigated in many papers. Some models focus on the size of infection, starting from the population models [6–8], whereas other focus on cell infection, starting from the virus models, which have become an important tool in both understanding HIV-1 infection in host and providing valuable insight into HIV pathogenesis. A well-known model for HIV infection is the following system [9, 10]:

1.1 $$ \textstyle\begin{cases} \frac{dx}{dt}=\lambda -dx-\beta xy, \\ \frac{dy}{dt}=\beta xy- ay, \\ \frac{dv}{dt}=ky-uv, \end{cases} $$

where variables *x*, *y*, and *v* represent the densities of the healthy cells, the infected cells, and the virus at time *t*, respectively. Here a mass action infection mechanism is adopted. The parameters *β* and *λ* stand for the infection and constant growth rates of the healthy cell, respectively. System (1.1) and its variations have been investigated in many papers [11–16]. Stephen et al. [17] recently added the term $\frac{\rho TV}{C+V}$ incorporating the homeostatic proliferation of T-cells, which leads to interesting dynamic results, such as bistability and Hopf bifurcation.

It is well-known that it takes a long period for an HIV to become an AIDS, and in the medical literature [18], it was pointed out that the latent reservoir (i.e., latent infection) was the main obstacle to eradicate the virus. Therefore the four-dimensional mathematical model including the latent infection seems more reasonable [19, 20]. In recent years, latent cells were considered in many models, such as Beddington–DeAngelis function response with delay [20], Crowley–Martin function response [21], and general infection function with CTC and VTC transmission [22].

To recover from a viral infection, the cytotoxic T lymphocyte (CTL), which can clear away the infected cells to prevent further viral replications, plays a particularly important role. In 1996, Nowak and Bangham [9] proposed the well-known model with immune response:

$$ \textstyle\begin{cases} \frac{dx}{dt}=\lambda -dx-\beta xy, \\ \frac{dy}{dt}=\beta xy- ay-pyz, \\ \frac{dv}{dt}=ky-uv, \\ \frac{dz}{dt}=cyz-bz, \end{cases} $$

where the variable *z* represents the concentration of CTLs. Many authors have studied the infective models with different immune responses, such as lytic and nonlytic immune responses [23, 24], cell-mediated immune mechanism or humoral immune mechanism [25–27], delayed immune response with drug therapies [28], and general CTL immune response with silent infected cell-to-cell spread [29].

However, after a viral infection, the CTLs that are responsible for clearing away the infected cells become cytotoxic T-lymphocyte precursors (CTLp) and have receptors for detecting the virus from the previous infection [30]. Upon contacting with the virus during a subsequent infection, the precursors differentiate and become cytotoxic T-lymphocyte effectors (CTLe), and these cells are again responsible for clearing away the invading virus. Considering this infective mechanism, Wodarz et al. [31, 32] provided the following model with CTL response:

1.2 $$ \textstyle\begin{cases} \frac{dx}{dt}=\lambda -dx-\beta xy, \\ \frac{dy}{dt}=\beta xy- ay-pyz, \\ \frac{dw}{dt}=cyw-cqyw-bw, \\ \frac{dz}{dt}=cqyw-hz, \end{cases} $$

where the healthy cells *x* and the infected cells *y* are described similarly as in system (1.1). Instead of just one class of CTL response, the CTLp and CTLe are introduced. The CTLp and CTLe are represented by *w* and *z*. These precursors emerge at rate $cyw$ and may become effectors at rate $cqyw$ or cleared away naturally at rate *bw*. Similarly, the effectors are created at rate $cqyw$ and cleared at rate *hz*.

In model (1.2), there is no virus term whose population is assumed at a quasi-steady state, which is proportional to infected cells. Model (1.2) is completely analyzed by Bernard et al. [33], who have found that the system transforms from one equilibrium to the next as the basic reproductive number $R_{0}$ increases. When $R_{0}$ increases further, they show that periodic solutions may arise from the third equilibrium via Hopf bifurcation.

In fact, another model with cytotoxic T-lymphocyte memory proposed in [31, 32] is given by

1.3 $$ \textstyle\begin{cases} \frac{dx}{dt}=\lambda -dx-\beta xy, \\ \frac{dy}{dt}=\beta xy- ay-pyz, \\ \frac{dw}{dt}=cxyw-cqyw-bw, \\ \frac{dz}{dt}=cqyw-hz. \end{cases} $$

This model assumes that the target cells are CD4^+^ T cells; moreover, it includes the additional feature that expansion of the CTLp population is proportional to both antigen and the number of uninfected CD4^+^ T-cells capable of delivering T-cell help. The memory generation depends on CD4^+^ T-cell help, and infection of CD4^+^ T-cells results in impaired T-cell help. We also assume that differentiation into effector functions is independent of CD4^+^ T-cell help [31]. A detailed explanation of the model can be found in [31]. All the parameters are positive.

Dynamics of system (1.3) is numerically analyzed in [31, 32]. In this paper, we provide a rigorous analytical method of system (1.3), and the basic framework is as follows. In Sect. 2, we establish the well-posedness of the model including nonnegativity and boundedness of the solutions, the existence of equilibria, and local stability of the boundary equilibria. The local stability analysis of the positive equilibria and their bifurcations are presented in Sect. 3. Numerical illustrations are given in Sect. 4. Finally, we discuss both mathematical and biological perspectives of the findings in Sect. 5.

## The equilibrium and stability of boundary equilibrium

For mathematical simplicity, we do some rescallings in system (1.3). Let $x=\sqrt{\frac{\lambda }{\beta }}\overline{x}$, $y=\sqrt{\frac{\lambda }{\beta }}\overline{y}$, $w=\frac{\beta }{pcq}\sqrt{\lambda \beta }\overline{w}$, $z=\frac{1}{p}\sqrt{\lambda \beta }\overline{z}$, $t=\frac{1}{\sqrt{\lambda \beta }}\tau $, $\overline{d}=\frac{d}{\sqrt{\lambda \beta }}$, $\overline{a}=\frac{a}{\sqrt{\lambda \beta }}$, $\overline{c}=\frac{c}{\beta }\sqrt{\frac{\lambda }{\beta }}$, $\overline{q}=\frac{cq}{\beta }$, $\overline{b}=\frac{b}{\sqrt{\lambda \beta }}$, and $\overline{h}=\frac{h}{\sqrt{\lambda \beta }}$. After changing back to the origin variables *x*, *y*, *w*, *z*, *t*, the scaled system is given by

2.1 $$ \textstyle\begin{cases} \frac{dx}{dt}=1-dx-xy, \\ \frac{dy}{dt}=xy- ay-yz, \\ \frac{dw}{dt}=cxyw-qyw-bw, \\ \frac{dz}{dt}=yw-hz, \end{cases} $$

where the horizontal lines on the heads of these parameters are removed, and the parameters *d̅*, *a̅*, *c̅*, *q̅*, *b̅*, *h̅* are replaced by *d*, *a*, *c*, *q*, *b*, *h*. Obviously, all the parameters are positive. The basic reproductive number of model (1.3) is $R_{00}=\frac{\lambda \beta }{ad}$, and for system (2.1), $R_{00}$ becomes $R_{0}=\frac{1}{ad}$.

### Theorem 2.1

*All solutions* $(x(t),y(t),w(t),z(t))$ *of system* (2.1) *are nonnegative for* $t>0$. *Moreover*, *if* $x(0)\geq 0$, $y(0)\geq 0$, $w(0)\geq 0$, *and* $z(0)\geq 0$, *then all solutions of system* (2.1) *are ultimately bounded*.

### Proof

By variation of constants we find the following solutions of (2.1):

2.2 $$ \textstyle\begin{cases} x(t)=x(0)e^{-\int _{0}^{t}(d+y(s))\,ds}+\int _{0}^{t}e^{-\int _{s}^{t}(d+y(u))\,du}\,ds, \\ y(t)=y(0)e^{\int _{0}^{t}(x(s)-a-z(s))\,ds}, \\ w(t)=w(0)e^{\int _{0}^{t}(x(s)y(s)-qy(s)-b)\,ds}, \\ z(t)=e^{-ht}(z(0)+\int _{0}^{t}y(s)w(s)e^{hs}\,ds), \end{cases} $$

which proves the nonnegativity of solutions of system (2.1).

Note that the first equation of (2.1) implies $\frac{dx}{dt}\leq 1-dx$. The solution is given by $x(t)\leq x(0)e^{-dt}+\frac{1}{d}$, which yields $\limsup_{t\rightarrow \infty }x(t)\leq \frac{1}{d}$. Adding the first two equations of (2.1), we obtain

$$ \frac{d(x+y)}{dt}=\frac{dx}{dt}+\frac{dy}{dt}=1-dx-ay-yz\leq 1-dx-ay \leq 1-d_{1}(x+y), $$

where $d_{1}=\min \{d,a\}$. It has the solution $x+y\leq (x(0)+y(0))e^{-d_{1}t}+\frac{1}{d_{1}}$, which implies $\limsup_{t\rightarrow \infty }(x(t)+y(t))\leq \frac{1}{d_{1}}$, and thus $x(t)$ and $y(t)$ are bounded.

Supposing that *z* is unbounded, by the second equation of (2.1) we have $\lim_{t\rightarrow \infty }y(t)=0$, which implies $\lim_{t\rightarrow \infty }w(t)=0$ from the third equation of (2.1). Then we get $\lim_{t\rightarrow \infty }z(t)=0$ from the fourth equation of (2.1), which contradicts with the unboundedness of *z*. Thus *z* must be bounded. Lastly, assume that *w* is unbounded. Based on the boundedness of *z* and the fourth equation of (2.1), we obtain $\lim_{t\rightarrow \infty }y(t)=0$, and from the third equation of (2.1) it follows that $\lim_{t\rightarrow \infty }w(t)=0$, which causes a contradiction. Hence *w* is bounded. The proof is complete. □

Theorem 2.1 shows that there exists a bounded positive invariant region $\Gamma \subset R_{+}^{4}$ for the system. Thus we concentrate on Γ to discuss the dynamics. In fact, the infection-free equilibrium $E_{0}=(\frac{1}{d}, 0, 0, 0)$ always exists, and there exists an infectious equilibrium without CTL, $E_{1}=(a, d(R_{0}-1), 0, 0)$ if $R_{0}>1$. To find the infectious equilibrium with CTL, it suffices to solve the system

2.3 $$ \textstyle\begin{cases} 1-dx-xy=0, \\ y=\frac{b}{cx-q}, \\ w=\frac{h z}{y}, \\ z=x-a. \end{cases} $$

After submitting *y*, *w*, *z* into the first equation of (2.3), we get the equation

2.4 $$ f(x):=c\, dx^{2}+(b-c-dq)x+q=0. $$

If $x_{1}$, $x_{2}$ are solutions of (2.4), then $x_{1}x_{2}=\frac{q}{cd}>0$ and $x_{1}+x_{2}=-\frac{b-c-dq}{cd}$. Hence, to ensure the existence of a positive solution, we have $b< c+dq$.

The determinant of (2.4) is

$$ \Delta =(b-c-dq)^{2}-4c dq . $$

Then $\Delta =0$ if and only if

$$ b=c+dq\pm 2\sqrt{c dq}. $$

Note that $c+dq-2\sqrt{c dq}\ge 0$. Combining this with the condition $b< c+dq$, we get that $\Delta \ge 0$ only if $0< b\le c+dq-2\sqrt{c dq}$, and $y>0$ and $z>0$ imply $x>\frac{q}{c}$ and $x>a$ by (2.3).

Set

2.5 $$ R_{1}=1+\frac{b}{d(ca-q)}. $$

Noting that $f(\frac{q}{c})=\frac{bq}{c}>0$, we obtain

2.6 $$ f(a)=a\, d(ca-q)\biggl[1+\frac{b}{d(ca-q)}-R_{0} \biggr]=\frac{ab}{R_{1}-1}(R_{1}-R_{0}). $$

### Notations

$x_{2\pm }=\frac{(c+dq-b)\pm \sqrt{(b-c-dq)^{2}-4c dq}}{2cd}$, $y_{2 \pm }=\frac{b}{cx_{2\pm }-q}$, $w_{2\pm }=\frac{h z_{2\pm }}{y_{2+}}$, $z_{2\pm }=x_{2\pm }-a$, $x_{20}=\frac{c+dq-b}{2cd}$, $y_{20}=\frac{2bd}{c-b-dq}$, $w_{20}=\frac{h(c+dq-b-2acd)(c-b-dq)}{4bcd^{2}}$, $z_{20}= \frac{c+dq-b-2acd}{2cd} $.

The positive equilibria of model (2.1) are classified by the sign of Δ. Let us consider three cases.

### Case I

$\Delta >0$, which is equivalent to $0< b< c+dq-2\sqrt{c dq}$. Here we consider three cases by the sign of $f(a)$.

1. $f(a)<0$. In this case, $\Delta >0$ is obviously satisfied, and $a>\frac{q}{c}$ is equivalent to $R_{1}>1$, and thus $R_{1}< R_{0}$ by (2.6). Combining this with $R_{1}>1$, we get that model (2.1) admits a unique positive equilibrium $E_{2+}=(x_{2+},y_{2+},w_{2+},z_{2+})$ if $1< R_{1}< R_{0}$.

2. $f(a)=0$. In this case, $R_{0}=R_{1}$, which indicates that *a* is a solution of $f(x)=0$, and the other root is $x_{*}=\frac{q}{acd}$. To get a positive equilibrium of model (2.1), $x_{*}=\frac{q}{acd}>a$ and $a>\frac{q}{c}$ are required, which yields $q>a^{2}cd$ and $ac>q$. Combining this with $\Delta >0$ (if and only if $0< b< c+dq-2\sqrt{c dq}$), model (2.1) admits a unique positive equilibrium $E_{2+}=(x_{2+},y_{2+},w_{2+},z_{2+})$ if $R_{1}=R_{0}>1$ and $a^{2}cd< q< ac$.

3. $f(a)>0$. Let us consider two cases.

(1) $\frac{q}{c}\geq a $, which is equivalent to $R_{1}\leq 1$. To ensure the positive equilibrium of model (2.1), the following conditions are required:

2.7 $$ \textstyle\begin{cases} f(\frac{q}{c})>0, \\ \Delta >0, \\ -\frac{b-c-dq}{2cd}>\frac{q}{c}, \\ \frac{q}{c}\geq a. \end{cases} $$

Note that $f(\frac{q}{c})>0$ always holds, and $\Delta >0$ if and only if $0< b< c+dq-2\sqrt{c dq}$, whereas $-\frac{b-c-dq}{2cd}>\frac{q}{c}$ if $0< b< c-dq$. Intersecting these two inequalities we have $c+dq-2\sqrt{c dq}< c-dq$ if $c>dq$, which is required to ensure that $b>0$. Note that $c\leq \frac{q}{a}$, Combining this with $c>dq$, we have $ad<1$ to ensure that the intersection is nonempty, and hence model (2.1) admits two positive equilibria $E_{2+}=(x_{2+},y_{2+},w_{2+},z_{2+})$ and $E_{2-}=(x_{2-},y_{2-},w_{2-},z_{2-})$ if $R_{0}>1\geq R_{1}$, $dq< c$, and $0< b< c+dq-2\sqrt{c dq}$.

(2) $\frac{q}{c}< a$, which is equivalent to $R_{1}>1$. Model (2.1) admits two positive equilibria if the following conditions are satisfied:

2.8 $$ \textstyle\begin{cases} f(a)>0, \\ \Delta >0, \\ -\frac{b-c-dq}{2cd}>a, \\ \frac{q}{c}< a. \end{cases} $$

Note that $f(a)>0$ if and only if $R_{1}>R_{0}$ by (2.6) and $R_{1}>1$, and $-\frac{b-c-dq}{2cd}>a$ is equivalent to $0< b< c+dq-2acd$; $\Delta >0$ if and only if $0< b< c+dq-2\sqrt{c dq}$. Note that $c+dq-2\sqrt{c dq}\leq c+dq-2acd$ if and only if $q\geq a^{2}cd$, whereas $c+dq-2\sqrt{c dq}>c+dq-2acd$ if and only if $q< a^{2}cd$, and hence model (2.1) admits two positive equilibria if one of the following conditions holds: (i) $R_{1}>1$, $R_{1}>R_{0}$, $q\geq a^{2}cd$, and $0< b< c+dq-2\sqrt{c dq}$; (ii) $R_{1}>1$, $R_{1}>R_{0}$, $q< a^{2}cd$, and $0< b< c+dq-2acd$.

### Case II

$\Delta =0$ if and only if $b=c+dq-2\sqrt{c dq}$, and model (2.1) admits a unique positive equilibrium if

2.9 $$ \textstyle\begin{cases} \Delta =0 , \\ -\frac{b-c-dq}{2cd}>a , \\ -\frac{b-c-dq}{2cd}>\frac{q}{c}. \end{cases} $$

Note that $-\frac{b-c-dq}{2cd}>a$ if $0< b< c+dq-2acd$, whereas $-\frac{b-c-dq}{2cd}>\frac{q}{c}$ yields $0< b< c-dq$. Combining these two conditions with $\Delta =0$ which is equivalent to $b=c+dq-2\sqrt{c dq}$, the following results are obtained: $c+dq-2\sqrt{c dq}< c+dq-2acd$ if $q>a^{2}cd$,while $c+dq-2\sqrt{c dq}< c-dq$ if $c>dq$, therefore model (2.1) admits a unique positive equilibrium if $b=c+dq-2\sqrt{c dq}$, $q>a^{2}cd$, and $c>dq$.

### Case III

If $\Delta <0$, then there is no positive equilibrium, because $f(x)=0$ has no real root. The following theorem summarizes all positive equilibria of system (2.1).

### Theorem 2.2

(1) *Model* (2.1) *admits a unique positive equilibrium* $E_{2+}=(x_{2+},y_{2+},w_{2+},z_{2+})$ *if one of the following conditions is satisfied*: (i) $1< R_{1}< R_{0}$; (ii) $R_{1}=R_{0}>1$ *and* $a^{2}cd< q< ac$.

(2) *Model* (2.1) *admits two positive equilibria* $E_{2+}=(x_{2+},y_{2+},w_{2+},z_{2+})$ *and* $E_{2-}=(x_{2-},y_{2-}, w_{2-},z_{2-})$ *if one of the following conditions is satisfied*: (i) $R_{0}>1\geq R_{1}$, $dq< c$, *and* $0< b< c+dq-2\sqrt{c dq}$; (ii) $R_{1}>1$, $R_{1}>R_{0}$, $q\geq a^{2}cd$, *and* $0< b< c+dq-2\sqrt{c dq}$; (iii) $R_{1}>1$, $R_{1}>R_{0}$, $q< a^{2}cd$, *and* $0< b< c+dq-2acd$.

(3) *Model* (2.1) *admits a unique positive equilibrium* $E_{20}=(x_{20},y_{20},w_{20},z_{20})$ *if* $b=c+dq-2\sqrt{c dq}$, $q>a^{2}cd$, *and* $c>dq$.

Next, we discuss the local stability and global stability of the boundary equilibria. The stability of the equilibria is based on the Jacobian matrix of model (2.1):

2.10 $$ J(x,y,w,z)= \begin{pmatrix} -d-y&-x&0&0 \\ y&x-a-z&0&-y \\ cyw&cxw-qw&cxy-qy-b & 0 \\ 0&w&y&-h \end{pmatrix}. $$

The characteristic roots at the equilibrium $E_{0}$ are given by

$$ s_{1}=-d< 0, \qquad s_{2}=-b< 0, \qquad s_{3}=-h< 0,\qquad s_{4}=\frac{1-da}{d}, $$

and $s_{4}=\frac{1-da}{d}=a(R_{0}-1)<0$ is equivalent to $R_{0}<1$. Hence the local stability of equilibrium $E_{0}$ is given by the following theorem.

### Theorem 2.3

*The infection*-*free equilibrium* $E_{0}$ *is asymptotically stable if* $R_{0}<1$ *and is unstable if* $R_{0}>1$.

### Theorem 2.4

*If* $R_{0}<1$, *then the infection*-*free equilibrium* $E_{0}$ *is globally asymptotically stable*.

### Proof

To show the global stability of equilibrium $E_{0}$, we use the method of fluctuation lemma employed by Hirsch et al. [34–36]. We introduce some notations. For a continuous bounded function $g:[0,\infty ]\rightarrow R$, let

$$ g^{\infty }=\limsup_{t\rightarrow \infty }g(t),\qquad g_{\infty }= \liminf_{t\rightarrow \infty }g(t), $$

By Theorem 2.1 the solutions $x(t)$, $y(t)$, $w(t)$, and $z(t)$ are always nonnegative and bounded for any nonnegative initial conditions, and the limits $\limsup_{t\rightarrow \infty }$ and $\liminf_{t\rightarrow \infty }$ always exist for each of these solutions. By the fluctuation lemma there exists a sequence $t_{n}$ such that

$$ \lim_{n\rightarrow \infty }x(t_{n})=x^{\infty },\qquad \lim _{n\rightarrow \infty } \dot{x}(t_{n})=0. $$

Set $t=t_{n}$. Then the first equation of model (2.1) gives $\dot{x}(t_{n})+dx(t_{n})+x(t_{n})y(x_{n})=1 $. Letting $n\rightarrow \infty $, it follows that

$$ d\lim_{n\rightarrow \infty }x(t_{n})+\lim_{n\rightarrow \infty }x(t_{n}) \lim_{n\rightarrow \infty }y(t_{n})=1, $$

which yields

2.11 $$ dx^{\infty }\leq dx^{\infty }+dx^{\infty }y_{\infty }=dx^{\infty }(1+y_{ \infty }) \leq dx^{\infty }\bigl(1+y^{\infty }\bigr)=1. $$

By a similar argument, for the remaining three equations in model (2.1), we get

2.12 $$\begin{aligned}& ay^{\infty }\leq y^{\infty }(a+z_{\infty }) \leq x^{\infty }y^{\infty }, \end{aligned}$$

2.13 $$\begin{aligned}& bw^{\infty }\leq w^{\infty }(b+qy_{\infty }) \leq cx^{\infty }y^{\infty }w^{ \infty }, \end{aligned}$$

2.14 $$\begin{aligned}& hz^{\infty }\leq y^{\infty }w^{\infty }. \end{aligned}$$

We claim that $y^{\infty }=0$. Otherwise, $y^{\infty }>0$; then it follows from (2.11) and (2.12) that $ay^{\infty }\leq x^{\infty }y^{\infty }\leq \frac{y^{\infty }}{d}$, that is, $(a-\frac{1}{d})y^{\infty }=a(1-R_{0})y^{\infty }\leq 0$. Therefore $R_{0}\geq 1$, which contradicts the condition $R_{0}<1$, and thus $y^{\infty }=0$. And from equations (2.13) and (2.14) we get that $w^{\infty }=0$ and $z^{\infty }=0$. The conditions $y^{\infty }=0$, $w^{\infty }=0$, and $z^{\infty }=0$ imply that $y(t)\rightarrow 0$, $w(t)\rightarrow 0$, and $z(t)\rightarrow 0$ as $t\rightarrow \infty $, respectively. Thus from the first equation of model (2.1) we have the asymptotic differential equation $\dot{x}=1-dx$. By simple calculation we get the solution $x(t)=x(0)e^{-dt}+\frac{1}{d}$, which clearly shows that the solution $x(t)\rightarrow \frac{1}{d}$ as $t\rightarrow \infty $ by the theory of asymptotically autonomous systems. The proof is complete. □

The characteristic equation of model (2.1) at $E_{1}$ is

2.15 $$ \bigl[(s+d+y_{1}) (s-x_{1}+a)+x_{1}y_{1} \bigr](s-cx_{1}y_{1}+qy_{1}+b) (s+h)=0. $$

Letting $(s+d+y_{1})(s-x_{1}+a)+x_{1}y_{1}=0$, we have $s^{2}+(d+y_{1})s+ay_{1}=0$. Note that $s_{1}+s_{2}=-(d+y_{1})<0$ and $s_{1}s_{2}=ay_{1}>0$, which imply that $s_{1}$ and $s_{2}$ have negative real parts. Obviously, $s_{3}=-h$ has a negative real part. In order for all the roots of equation (2.15) to have negative real parts, it is required that

$$ s_{4}=cx_{1}y_{1}-qy_{1}-b=d(R_{0}-1) (ca-q)-b= \frac{b(R_{0}-R_{1})}{R_{1}-1}< 0, $$

which implies $R_{1}>R_{0}>1$ or $R_{0}>1>R_{1}$.

### Theorem 2.5

*Equilibrium* $E_{1}$ *is locally asymptotically stable for* $1< R_{0}< R_{1}$ *or* $R_{0}>1>R_{1}$ *and is unstable for* $1< R_{1}< R_{0}$.

### Remark

Note that $f(a)=\frac{ab}{(R_{1}-1)}(R_{1}-R_{0})=-as_{4}$, from which it follows that $E_{1}$ is locally asymptotically stable, whereas system (2.1) may have positive equilibrium under certain conditions by Theorem 2.2 (case I.3). Moreover, system (2.1) may have both stable positive equilibrium and stable boundary equilibrium $E_{1}$.

## Stability of positive equilibria and their bifurcations

By an easy calculation the characteristic equation of the positive equilibrium follows:

3.1 $$ s^{4}+\alpha _{1}s^{3}+ \alpha _{2}s^{2}+\alpha _{3}s+\alpha _{4}=0, $$

where

3.2 $$ \textstyle\begin{cases} \alpha _{1}=h+\frac{1}{x}, \\ \alpha _{2}=xy+\frac{h}{x}+yw, \\ \alpha _{3}=hxy+\frac{yw}{x}+byw, \\ \alpha _{4}=\frac{byw}{x}-cwxy^{3}. \end{cases} $$

Here *x*, *y*, *w*, *z* are the coordinates of the positive equilibria. Obviously, $\alpha _{1}>0$, $\alpha _{2}>0$, and $\alpha _{3}>0$, and we only need to judge the sign of $\alpha _{4}$.

Firstly, we focus on the positive equilibrium $E_{20}$. By calculation we have

$$ \alpha _{4}=\frac{by_{20}w_{20}}{x_{20}}-cw_{20}x_{20}y_{20}^{3}= \frac{w_{20}y_{20}(b-cx_{20}^{2}y_{20}^{2})}{x_{20}}. $$

Note that $1-dx_{20}-x_{20}y_{20}=0$, and it follows that

3.3 $$ b-cx_{20}^{2}y_{20}^{2}=-cd^{2}x_{20}^{2}+2cdx_{20}+b-c= \frac{4dcq-(c+dq-b)^{2}}{4c}=-\frac{\Delta }{4c}=0, $$

which indicates that $\alpha _{4}=0$, and the characteristic equation of equilibrium $E_{20}$ becomes

$$ s\bigl(s^{3}+\alpha _{1}s^{2}+\alpha _{2}s+\alpha _{3}\bigr)=0. $$

It has a characteristic root $s_{1}=0$, and $s_{2}$, $s_{3}$, $s_{4}$ are determined by the equation

3.4 $$ s^{3}+\alpha _{1}s^{2}+ \alpha _{2}s+\alpha _{3}=0, $$

where

3.5 $$ \textstyle\begin{cases} \alpha _{1}=h+\frac{1}{x_{20}}>0, \\ \alpha _{2}=x_{20}y_{20}+\frac{h}{x_{20}}+y_{20}w_{20}>0, \\ \alpha _{3}=hx_{20}y_{20}+\frac{y_{20}w_{20}}{x_{20}}+by_{20}w_{20}>0. \end{cases} $$

Note that $\alpha _{i}>0$, $i=1, 2, 3$, and according to the Routh–Hurwitz criterion, $s_{2}$, $s_{3}$, and $s_{4}$ have negative real parts only if $\Delta _{20}=\alpha _{1}\alpha _{2}-\alpha _{3}=\frac{h^{2}}{x_{20}}+y_{20}+ \frac{h}{x_{20}^{2}}+(h-b)y_{20}w_{20}>0$.

Next, we focus on $s_{1}=0$. Transforming the equilibrium $E_{20}$ to the origin by $\overline{x}=x-x_{20}$, $\overline{y}=y-y_{20}$, $\overline{w}=w-w_{20}$, $\overline{z}=z-z_{20}$, system (2.1) becomes

3.6 $$ \textstyle\begin{cases} \frac{dx}{dt}=-\frac{1}{x_{20}}x-x_{20}y-xy, \\ \frac{dy}{dt}=y_{20}x-y_{20}z+xy-yz, \\ \frac{dw}{dt}=cxyw+cw_{20}xy+cy_{20}xw+cy_{20}w_{20}x+ \frac{byw}{y_{20}}+\frac{bw_{20}y}{y_{20}}, \\ \frac{dz}{dt}=y_{20}w+w_{20}y-hz+yw, \end{cases} $$

where the horizontal lines on the heads of these letters are removed, and we still denote *x̅*, *y̅*, *w̅*, *z̅* by *x*, *y*, *w*, *z*. Set

$$ u= \begin{pmatrix} 1&0&0&0 \\ 0&1&0&0 \\ \frac{bw_{20}}{x_{20}y_{20}}&0&1& 0 \\ 0&0&0&1 \end{pmatrix}. $$

Under the transformations $(\overline{x}, \overline{y}, \overline{w}, \overline{z})^{T}=u(x, y, w, z)^{T}$, model (3.6) becomes

3.7 $$ \textstyle\begin{cases} \frac{dx}{dt}=-\frac{1}{x_{20}}x-x_{20}y-xy, \\ \frac{dy}{dt}=y_{20}x-y_{20}z+xy-yz, \\ \frac{dw}{dt}=-\frac{bcw_{20}}{x_{20}}x^{2}+(\frac{qw_{20}}{x_{20}}- \frac{b^{2}w_{20}}{x_{20}y_{20}^{2}})xy+ cy_{20}xw+\frac{b}{y_{20}}yw+cxyw- \frac{bcw_{20}}{x_{20}y_{20}}x^{2}y, \\ \frac{dz}{dt}=y_{20}w+w_{20}y-hz+yw-\frac{bw_{20}}{x_{20}}x- \frac{bw_{20}}{x_{20}y_{20}}xy, \end{cases} $$

where the horizontal lines on the heads of these letters are removed, and we still denote *x̅*, *y̅*, *w̅*, *z̅* by *x*, *y*, *w*, *z*. The third equation of model (3.7) has no linear term, and then the center manifold is a curve tangent to the *w*-axis.

To obtain an approximative expression of the center manifold, we set

3.8 $$ \textstyle\begin{cases} x=m_{1}w+m_{2}w^{2}+o(w^{2}), \\ y=n_{1}w+n_{2}w^{2}+o(w^{2}), \\ z=p_{1}w+p_{2}w^{2}+o(w^{2}), \end{cases} $$

where $m_{1}$, $n_{1}$, $p_{1}$, $m_{2}$, $n_{2}$, $p_{2}$ are undetermined coefficients. It follows that

3.9 $$ \textstyle\begin{cases} \frac{dx}{dt}=m_{1}\frac{dw}{dt}+[2m_{2}w+o(w)]\frac{dw}{dt}, \\ \frac{dy}{dt}=n_{1}\frac{dw}{dt}+[2n_{2}w+o(w)]\frac{dw}{dt}, \\ \frac{dz}{dt}=p_{1}\frac{dw}{dt}+[2p_{2}w+o(w)]\frac{dw}{dt}. \end{cases} $$

To find the unknown coefficients $m_{1}$, $m_{2}$, $n_{1}$, $n_{2}$, $p_{1}$, $p_{2}$, we substitute (3.7) and (3.8) into (3.9), compare the coefficients at *w* and $w^{2}$, and obtain

3.10 $$ \textstyle\begin{cases} \frac{1}{x_{20}}m_{1}+n_{1}x_{20}=0, \\ -(\frac{m_{2}}{x_{20}}+n_{2}x_{20}+m_{1}n_{1}) \\ \quad =[- \frac{bcm_{1}^{2}w_{20}}{x_{20}} +(\frac{qw_{20}}{x_{20}}- \frac{b^{2}w_{20}}{x_{20}y_{20}^{2}})m_{1}n_{1}+cm_{1}y_{20}+ \frac{b}{y_{20}}n_{1}]m_{1}, \\ y_{20}m_{1}-y_{20}p_{1}=0, \\ y_{20}m_{2}-y_{20}p_{2}+m_{1}n_{1}-n_{1}p_{1} \\ \quad =n_{1}[- \frac{bcm_{1}^{2}w_{20}}{x_{20}} +(\frac{qw_{20}}{x_{20}}- \frac{b^{2}w_{20}}{x_{20}y_{20}^{2}})m_{1}n_{1}+cm_{1}y_{20}+ \frac{b}{y_{20}}n_{1}], \\ y_{20}+n_{1}w_{20}-hp_{1}-\frac{bw_{20}}{x_{20}}m_{1}=0, \\ n_{2}w_{20}-hp_{2}+n_{1}-\frac{bw_{20}m_{2}}{x_{20}}- \frac{bw_{20}m_{1}n_{1}}{x_{20}y_{20}} \\ \quad =p_{1}[- \frac{bcm_{1}^{2}w_{20}}{x_{20}} +(\frac{qw_{20}}{x_{20}}- \frac{b^{2}w_{20}}{x_{20}y_{20}^{2}})m_{1}n_{1} +cm_{1}y_{20}+\frac{b}{y_{20}}n_{1}]. \end{cases} $$

According to (3.8) and (3.9), we have

3.11 $$ \frac{dw}{dt}=\biggl[-\frac{bcm_{1}^{2}w_{20}}{x_{20}} +\biggl( \frac{qw_{20}}{x_{20}}-\frac{b^{2}w_{20}}{x_{20}y_{20}^{2}}\biggr)m_{1}n_{1}+cm_{1}y_{20}+ \frac{b}{y_{20}}n_{1}\biggr]w^{2}+o \bigl(w^{2}\bigr). $$

Note that $\frac{dw}{dt}$ is only related to $m_{1}$, $n_{1}$, and $p_{1}$, and it follows that (by (3.10))

$$\begin{aligned}& m_{1}=\frac{y_{20}x_{20}^{2}}{hx_{20}^{2}+w_{20}+bw_{20}x_{20}}, \qquad p_{1}= \frac{y_{20}x_{20}^{2}}{hx_{20}^{2}+w_{20}+bw_{20}x_{20}}, \\& n_{1}=- \frac{y_{20}}{hx_{20}^{2}+w_{20}+bw_{20}x_{20}}. \end{aligned}$$

Substituting $m_{1}$, $p_{1}$, and $n_{1}$ into (3.11) and noting that $b-cx_{20}^{2}y_{20}^{2}=0$, we have

$$\begin{aligned} \frac{dw}{dt}&=\biggl( \frac{bw_{20}x_{20}(b-cx_{20}^{2}y_{20}^{2})-qw_{20}x_{20}y_{20}^{2}}{(w_{20}+hx_{20}^{2}+bw_{20}x_{20})^{2}} +\frac{cx_{20}^{2}y_{20}^{2}-b}{w_{20}+hx_{20}^{2}+bw_{20}x_{20}} \biggr)w^{2}+o\bigl(w^{2}\bigr) \\ &=- \frac{qw_{20}x_{20}y_{20}^{2}}{(w_{20}+hx_{20}^{2}+bw_{20}x_{20})^{2}}w^{2}+o\bigl(w^{2}\bigr). \end{aligned}$$

The coefficient of $w^{2}$ is $- \frac{qw_{20}x_{20}y_{20}^{2}}{(w_{20}+hx_{20}^{2}+bw_{20}x_{20})^{2}}<0$. Hence the equilibrium $E_{20}$ is a saddle-node.

### Theorem 3.1

*If* $\frac{h^{2}}{x_{20}}+y_{20}+\frac{h}{x_{20}^{2}}+(h-b)y_{20}w_{20}>0$, *then the infectious equilibrium* $E_{20}$ *is a saddle*-*node*.

For the equilibria $E_{2-}$ and $E_{2+}$, we have the following properties.

### Proposition 3.1

*When the equilibria* $E_{2-}$ *and* $E_{2+}$ *exist*, *then* $\det (J(E_{2-}))<0$ *and* $\det (J(E_{2+}))>0$.

### Proof

Similarly to the analysis of equilibrium $E_{20}$, we obtain $\alpha _{4}=\frac{byw}{x}-cwxy^{3}=\frac{yw}{x}(b-cx^{2}y^{2})$. Set

$$ g(x)\doteq b-cx^{2}y^{2}=-cd^{2}x^{2}+2cdx+b-c, $$

which is strictly increasing in $(-\infty ,\frac{1}{d})$. Suppose $x_{2+}<\frac{1}{d}$. Submitting $x_{2+}$ into this inequality and simplifying it, we have $c+b-dq>\sqrt{\Delta }$. Note that $c+b-dq>0$. Squaring both sides and simplifying, we obtain $4bc>0$, which implies $x_{2-}<\frac{c+dq-b}{2cd}<x_{2+}<\frac{1}{d}$.

An easy calculation shows that $g(\frac{c+dq-b}{2cd})=-\frac{\Delta }{4c}$ by (3.3). Then $g(\frac{c+dq-b}{2cd})<0$ when $\Delta >0$. It follows that $g(x_{2-})< g(\frac{c+dq-b}{2cd})<0$, which yields $\det (J(E_{2-}))=\alpha _{4}=\frac{y_{2-}w_{2-}}{x_{2-}}g(x_{2-})<0$.

Solving $g(x)=0$, we obtain the roots $x_{+}=\frac{c+\sqrt{bc}}{cd}$, $x_{-}=\frac{c-\sqrt{bc}}{cd}$. In fact, $x_{-}<\frac{1}{d}<x_{+}$. Suppose $x_{-}< x_{2+}$, that is, $\frac{c+dq-b+\sqrt{\Delta }}{2cd}>\frac{c-\sqrt{bc}}{cd}$. By direct calculation we have $\sqrt{\Delta }>c+b-dq-2\sqrt{bc}$. If $c+b-dq-2\sqrt{bc}\leq 0$, then it holds. If $c+b-dq-2\sqrt{bc}>0$, after squaring both sides and simplifying, we obtain $(b-c-dq)^{2}-(c+b-dq)^{2}>4dcq+4bc-4\sqrt{bc}(c+b-dq)$, which gives $b+c-dq-2\sqrt{bc}>0$. It follows that $\frac{1}{d}>x_{2+}>x_{-}$, and hence $g(x_{2+})>g(x_{-})=0 $, which in turn yields $\det (J(E_{2+}))=\alpha _{4}=\frac{y_{2+}w_{2+}}{x_{2+}}g(x_{2+})>0$. This completes the proof. □

### Theorem 3.2

*The infectious equilibrium* $E_{2-}$ *is unstable once it exists*.

### Proof

Since the determinant $\det (E_{2-})<0$, there is at least one characteristic root that has no negative real part. Therefore $E_{2-}$ is unstable. □

Next, we discuss the stability of equilibria $E_{2+}$. By (3.2) the characteristic equation at $E_{2+}$ is given by

$$ s^{4}+\alpha _{1}s^{3}+\alpha _{2}s^{2}+\alpha _{3}s+\alpha _{4}=0, $$

where

$$ \textstyle\begin{cases} \alpha _{1}=h+\frac{1}{x_{2+}}, \\ \alpha _{2}=x_{2+}y_{2+}+\frac{h}{x_{2+}}+y_{2+}w_{2+}, \\ \alpha _{3}=hx_{2+}y_{2+}+\frac{y_{2+}w_{2+}}{x_{2+}}+by_{2+}w_{2+}, \\ \alpha _{4}=\frac{by_{2+}w_{2+}}{x_{2+}}-cw_{2+}x_{2+}y_{2+}^{3}. \end{cases} $$

Note that $\alpha _{1}>0$, $\alpha _{2}>0$, and $\alpha _{3}>0$, and according to the proof of Propositiony 3.1, we know that $\alpha _{4}>0$. The relevant Routh–Hurwitz determinants are

3.12 $$ \textstyle\begin{cases} \Delta _{1}=\alpha _{1}, \\ \Delta _{2}=\alpha _{1}\alpha _{2}-\alpha _{3}, \\ \Delta _{3}=\alpha _{3}\Delta _{2}-\alpha _{1}^{2}\alpha _{4}, \\ \Delta _{4}=\alpha _{4}\Delta _{3}. \end{cases} $$

Note that $\Delta _{1}>0$ and the sign of $\Delta _{4}$ is the same as $\Delta _{3}$. By the formulas of $x_{2+}$, $y_{2+}$, and $z_{2+}$, $\Delta _{2}$ and $\Delta _{3}$ can be written more explicitly as

3.13 $$ \textstyle\begin{cases} \Delta _{2}=A_{2}h^{2}+B_{2}h+C_{2}, \\ \Delta _{3}=\frac{h}{x_{2+}^{3}}[A_{3}h^{2}+B_{3}h+C_{3}], \end{cases} $$

where

$$ \textstyle\begin{cases} A_{2}=\frac{1}{x_{2+}}+z_{2+}, \\ B_{2}=\frac{1}{x_{2+}^{2}}-bz_{2+}, \\ C_{2}=y_{2+}, \\ A_{3}=x_{2+}^{3}y_{2+}+x_{2+}z_{2+}+x_{2+}^{4}y_{2+}z_{2+}+x_{2+}^{2}z_{2+}^{2}+bz_{2+}^{2}x_{2+}^{3}+cz_{2+}x_{2+}^{4}y_{2+}^{2}, \\ B_{3}=x_{2+}^{2}y_{2+}+z_{2+}+2cx_{2+}^{3}z_{2+}y_{2+}^{2}-bx_{2+}^{4}y_{2+}z_{2+}-bx_{2+}^{2}z_{2+}^{2}-b^{2}z_{2+}^{2}x_{2+}^{3}-bz_{2+}x_{2+}, \\ C_{3}=x_{2+}^{4}y_{2+}^{2}+x_{2+}^{2}y_{2+}z_{2+}+bx_{2+}^{3}y_{2+}z_{2+}+cx_{2+}^{2}z_{2+}y_{2+}^{2}-bz_{2+}. \end{cases} $$

Next, we give the following lemma to show that if both $\Delta _{2}$ and $\Delta _{3}$ can become zero, then $\Delta _{3}$ will cross zero before $\Delta _{2}$ does.

### Lemma 3.1

*If* $E_{2+}$ *exists*, *then* $\Delta _{2}$ *is positive when* $\Delta _{3}$ *crosses zero for some change in parameters*.

### Remark

The proof is similar to that in [33], so here we omit it.

Thus, to consider the stability of the positive equilibrium $E_{2+}$, we only need to consider the possibility of $\Delta _{3}=0$ (see [33]). Note that *x*, *y*, *z* do not contain *h* and $\Delta _{3}=0$ is just a quadratic equation in terms of *h*. Let

3.14 $$ \textstyle\begin{cases} \Delta _{4}=B_{3}^{2}-4A_{3}C_{3}, \\ h_{2}^{*}=\frac{-B_{3}+\sqrt{\Delta _{4}}}{2A_{3}}, \\ h_{1}^{*}=\frac{-B_{3}-\sqrt{\Delta _{4}}}{2A_{3}}, \\ h^{*}=-\frac{B_{3}}{2A_{3}}. \end{cases} $$

Obviously, $h_{1}^{*}< h^{*}< h_{2}^{*}$, and thus we have the following theorem.

### Theorem 3.3

*Suppose the infectious equilibrium* $E_{2+}$ *exists*.

(1) *If* $C_{3}<0$, *then* $E_{2+}$ *is locally asymptotically stable when* $h\in (h_{2}^{*},+\infty )$ *and is unstable when* $h\in (0,h_{2}^{*})$.

(2) *If* $C_{3}>0$, *then*

(i) $E_{2+}$ *is always locally asymptotically stable when* $B_{3}\geq 0$, *and*

(ii) *if* $B_{3}<0$, *then* $E_{2+}$ *is always locally asymptotically stable when* $\Delta _{4}<0$. *Moreover*, *if* $\Delta _{4}=0$, *then* $E_{2+}$ *is locally asymptotically stable when* $h\neq h^{*}$; *if* $\Delta _{4}>0$, *then* $E_{2+}$ *is locally asymptotically stable when* $h\in (0,h_{1}^{*})\cup (h_{2}^{*},+\infty )$ *and unstable when* $h\in (h_{1}^{*}, h_{2}^{*})$.

(3) *If* $C_{3}=0$, *then* $E_{2+}$ *is always locally asymptotically stable when* $B_{3}\geq 0$, *and if* $B_{3}<0$, *then* $E_{2+}$ *is locally asymptotically stable when* $h\in (-\frac{B_{3}}{A_{3}}, +\infty )$ *and unstable when* $h\in (0, -\frac{B_{3}}{A_{3}})$.

### Remarks

1. The proof is straightforward by considering the sign of the quadratic polynomial $A_{3}h^{2} + B_{3}h + C_{3}$, and the detailed proof is contained in Theorem 3.4, so we omit it.

2. If we use the computing method of paper [33], then $\Delta _{2}$ and $\Delta _{3}$ can be written more explicitly as

3.15 $$ \textstyle\begin{cases} \Delta _{2}=A_{22}(h-b)^{2}+B_{22}(h-b)+C_{22}, \\ \Delta _{3}=A_{33}(h-b)^{2}+B_{33}(h-b)+C_{33}, \end{cases} $$

where

$$ \textstyle\begin{cases} A_{22}=\frac{1}{x_{2+}}+z_{2+}, \\ B_{22}=\frac{2b}{x_{2+}}+bz_{2+}+\frac{1}{x_{2+}^{2}}, \\ C_{22}=\frac{b^{2}}{x_{2+}}+\frac{b}{x_{2+}^{2}}+y_{2+}, \\ A_{33}=\frac{1}{x_{2+}}(x_{2+}y_{2+}+\frac{z_{2+}}{x_{2+}})+z_{2+}(x_{2+}y_{2+}+ \frac{z_{2+}}{x_{2+}}+bz_{2+})+ \frac{cz_{2+}x_{2+}^{2}y_{2+}^{2}}{x_{2+}}, \\ B_{33}=2by_{2+}+(\frac{1}{x_{2+}^{2}+bz_{2+}})(x_{2+}y_{2+}+ \frac{z_{2+}}{x_{2+}}+bz_{2+})+2bcz_{2+}x_{2+}y_{2+}^{2}+2cz_{2+}y_{2+}^{2}, \\ C_{33}=b^{2}y_{2+}+\frac{by_{2+}}{x_{2+}}+y_{2+}(x_{2+}y_{2+}+ \frac{z_{2+}}{x_{2+}}+bz_{2+})+cx_{2+}^{2}y_{2+}^{2} ( \frac{b^{2}z_{2+}}{x_{2+}}+\frac{2bz_{2+}}{x_{2+}^{2}}+ \frac{z_{2+}}{x_{2+}^{3}}). \end{cases} $$

Obviously, $A_{22}>0$, $B_{22}>0$, $C_{22}>0$, $A_{33}>0$, $B_{33}>0$, and $C_{33}>0$, which indicates that $\Delta _{22}>0$ and $\Delta _{33}>0$ as long as $h>b$. As in [33], the infectious equilibrium $E_{2+}$ with CTL response is always stable if the death rate of the CTLe is higer than that of the CTLp.

### Theorem 3.4

*Consider the infectious equilibrium* $E_{2+}$(*here* $E_{2+}$ *exists*).

(1) *If* $C_{3}<0$, *then a Hopf bifurcation occurs at* $h=h_{2}^{*}$.

(2) *If* $C_{3}>0$, $B_{3}<0$, *and* $\Delta _{4}>0$, *then two Hopf bifurcations occur at* $h=h_{1}^{*}$ *and* $h=h_{2}^{*}$.

(3) *If* $C_{3}=0$ *and* $B_{3}<0$, *then a Hopf bifurcation occurs at* $h=-\frac{B_{3}}{A_{3}}$.

### Proof

As in [37], a four-dimensional model has a Hopf bifurcation if $\Delta _{1}>0$, $\Delta _{2}>0$, and $\Delta _{3}=0$. Obviously, $\Delta _{1}>0$ always holds by (3.12), and according to Lemma 3.1, $\Delta _{2}>0$ as $\Delta _{3}$ crosses zero for some change of parameters. Therefore a Hopf bifurcation occurs as $\Delta _{3}=0$, which is equivalent to finding the roots of the quadratic polynomial equation $A_{3}h^{2}+B_{3}h+C_{3}=0$, where $A_{3}$, $B_{3}$, $C_{3}$ are independent of *h* by (3.13) and (3.14). Hence $A_{3}h^{2}+B_{3}h+C_{3}=0$ is a quadratic equation of *h*. Note that if $C_{3}<0$, then a positive root $h_{2}^{*}=\frac{-B_{3}+\sqrt{\Delta _{3}}}{2A_{3}}$ exists, which indicates that $E_{2+}$ is stable for $h \in (0,h_{2}^{*})$ and $E_{2+}$ is unstable for $h\in (h_{2}^{*}, +\infty )$, whereas $\Delta _{3}=0$ if $h=h_{2}^{*}$. Hence by [37] a Hopf bifurcation occurs at $h=h_{2}^{*}$.

However, if $C_{3}>0$ and $B_{3}<0$, then $\Delta _{3}>0$ as $h\in (0,h_{1}^{*})\cup (h_{2}^{*},+\infty )$, which implies that $E_{2+}$ is stable, whereas $\Delta _{3}<0$ as $h\in (h_{1}^{*}, h_{2}^{*})$, which ensures that $E_{2+}$ is unstable, and $\Delta _{3}=0$ as $h=h_{2}^{*}$ or $h=h_{1}^{*}$. Hence by [37] two Hopf bifurcations occur at $h=h_{2}^{*}$ and $h=h_{1}^{*}$.

If $C_{3}=0$ and $B_{3}<0$, then $\Delta _{3}>0$ as $h\in (-\frac{B_{3}}{A_{3}},+\infty )$, which implies that $E_{2+}$ is stable, whereas $\Delta _{3}<0$ as $h\in (0, -\frac{B_{3}}{A_{3}})$, which ensures that $E_{2+}$ is unstable, and $\Delta _{3}=0$ as $h=-\frac{B_{3}}{A_{3}}$. Thus by [37] a Hopf bifurcation occurs at $h=-\frac{B_{3}}{A_{3}}$. □

### Remark

Since the expressions of *x*, *y*, *w*, *z* are very complicated, it is too complicate to directly discuss the sign of $\Delta _{4}=B_{3}^{2}-4A_{3}C_{3}$. In the next section, by numerical calculations we will demonstrate that the three cases $\Delta _{4}>0$, $\Delta _{4}=0$, and $\Delta _{4}<0$ are possible.

## Numerical illustrations

In this section, we demonstrate the theoretical results by numerical simulations. For convenience, we will work on the scaled model (2.1) instead of the original model (1.3).

The values of *λ* used for simulations in [32] are $\lambda =1$ and $\lambda =10$, and as a bifurcation in [33], this indicates that the values of *λ* have a fairly large variation; therefore we take $\lambda =0.5657$. Note that $\beta =0.5$ and $\beta =0.001$ were used in [32], whereas $\beta =\frac{3}{400}$ was used in [33], and hence choosing $\beta =0.0707$ is reasonable. The parameter $q\in [0,1]$ represents the decomposition rate; here we choose $q=0.7071$. The same parameter values as in [32] are taken, and appropriate value for *h* is chosen:

$$ d=0.1,\qquad p=1,\qquad c=0.1,\qquad b=0.1,\qquad h=0.06. $$

According to the relationship between the parameters of the original model (1.3) and the scaled model (2.1), the parameter values in the scaled model (2.1) are chosen as follows:

$$ c=4,\qquad b=0.5,\qquad q=1,\qquad h=0.3,\qquad d=0.5. $$

Choose *a* as the bifurcation parameter. With these parameter values, we obtain

$$ R_{0}=\frac{1}{ad}=\frac{1}{0.5a}= \frac{2}{a}, \qquad R_{1}=1+ \frac{b}{d(ca-q)}=1+ \frac{0.5}{0.5(4a-1)}=1+\frac{1}{4a-1}. $$

**Figure 1:**
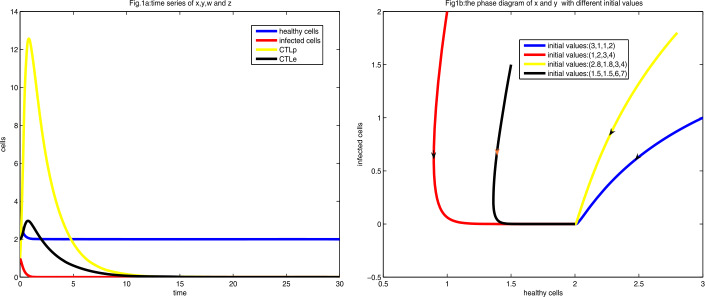
Figure 1(**a**) is the diagram of time series of *x*, *y*, *w*, and *z* at $a=3$ with the initial value $(3, 1, 1, 2)$. Figure 1(**b**) is the phase diagram of *x* and *y* with different initial values

To get $R_{0}<1$, $a>2$ is required, which in turn yields that the infection-free equilibrium $E_{0}=(\frac{1}{d}, 0, 0, 0)=(2, 0, 0, 0)$ is globally stable by Theorem 2.4. For $a=3$, system (2.1) has a globally stable infection-free equilibrium $E_{0}$, which is shown in Fig. 1.

As shown in Fig. 1, the healthy and infected cells decrease and converge to the equilibrium $E_{0}$ directly, whereas CTLe and CTLp firstly increase, then decrease, and finally converge to zero, which revels that the infected cells CTLp and CTLe die out directly after a brief fluctuation.

As *a* decreases to the critical value $a_{1}^{c}\doteq 2$, $R_{0}$ increases and passes the threshold 1, which reveals that $E_{0}$ becomes unstable. However, the infectious equilibrium $E_{1}=(a, d(R_{0}-1), 0, 0)=(a, 0.5(\frac{2}{a}-1), 0, 0)$ without CTL appears. As stated in Theorem 2.5, $E_{1}$ is stable if $1< R_{0}< R_{1}$ or $R_{0}>1>R_{1}$. By direct calculation we have $R_{0}< R_{1}$ if $2a^{2}-4a+1=(a-\frac{2+\sqrt{2}}{2})(a-\frac{2-\sqrt{2}}{2})>0$, which implies that $1< R_{0}< R_{1}$ is equivalent to $1.707\approx \frac{2+\sqrt{2}}{2}< a<2$ or $\frac{1}{4}< a<\frac{2-\sqrt{2}}{2}\approx 0.2930$, whereas $R_{0}>1>R_{1}$ is equivalent to $0< a<\frac{1}{4}$. Adding the second case together, we conclude that $E_{1}$ is stable if $a\in (0, \frac{1}{4}) \cup (\frac{1}{4}, \frac{2-\sqrt{2}}{2}) \cup ( \frac{2+\sqrt{2}}{2}, 2)$.

**Figure 2:**
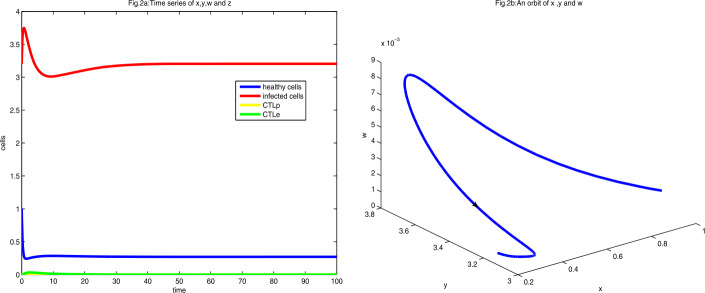
Figure 2(**a**) is the diagram of time series of *x*, *y*, *w*, and *z* at $a=0.27$ with initial value $(1, 3.2, 0.001, 0.002)$. Figure 2(**b**) is the phase diagram of *x*, *y*, and *z* with the same initial value

Let $a=0.27$. Obviously, $E_{1}=(a, d(R_{0}-1), 0, 0)=(0.27, \frac{173}{54}, 0, 0)$ is stable, as shown in Fig. 2.

As shown in Figs. 2(a) and 2(b), all cells converge to the equilibrium $E_{1}$ after a short time.

According to Theorem 2.2, system (2.1) admits a unique positive equilibrium

$$\begin{aligned} \begin{aligned} E_{2+}&=(x_{2+}, y_{2+}, w_{2+}, z_{2+}) \\ &=\biggl(1+\frac{1}{2}\sqrt{2}, \frac{3}{2}- \sqrt{2}, -\frac{3}{10}(3+2\sqrt{2}) (-2-\sqrt{2}+2a), 1+ \frac{1}{2}\sqrt{2}-a\biggr) \end{aligned} \end{aligned}$$

if $\frac{2-\sqrt{2}}{2}< a<\frac{2+\sqrt{2}}{2}$ (Theorem 2.2 (1)(i)). If $a \in (0, \frac{1}{4})\cup (\frac{1}{4}, \frac{2-\sqrt{2}}{2})$ (Theorem 2.2 (2)(i),2(ii)), then system (2.1) admits two positive equilibria $E_{2+}=(x_{2+}, y_{2+}, w_{2+}, z_{2+})$ and

$$ E_{2-}=\biggl(1-\frac{1}{2}\sqrt{2}, \frac{3}{2}+ \sqrt{2}, \frac{3}{10}(-3+2 \sqrt{2}) (-2+\sqrt{2}+2a), 1- \frac{1}{2}\sqrt{2}-a\biggr), $$

which is unstable once it exists.

Next, we will prove Theorem 3.3 numerically. By direct calculation the characteristic equation at $E_{2+}$ in term of *a* is (here $h=0.3$ is a constant)

$$ P(s)=s^{4}+\alpha _{1}s^{3}+\alpha _{2}s^{2}+\alpha _{3}s+\alpha _{4}=0, $$

where

4.1 $$ \textstyle\begin{cases} \alpha _{1}=\frac{23}{10}-\sqrt{2}, \\ \alpha _{2}=\frac{7}{5}-\frac{2}{5}\sqrt{2}-\frac{3}{10}a, \\ \alpha _{3}=\frac{3}{340}(-5+2\sqrt{2})(-20+17a-8\sqrt{2}), \\ \alpha _{4}=-\frac{3}{20}(-4+3\sqrt{2})(-2-\sqrt{2}+2a). \end{cases}$$

It follows that the existence conditions of positive equilibrium $E_{2+}$ ($a\in (0,\frac{1}{4})\cup (\frac{1}{4},\frac{2-\sqrt{2}}{2})$ or $a\in (\frac{2-\sqrt{2}}{2},\frac{2+\sqrt{2}}{2})$) directly guarantee that $\alpha _{i}>0$, $i=1,2,3,4$.

**Figure 3:**
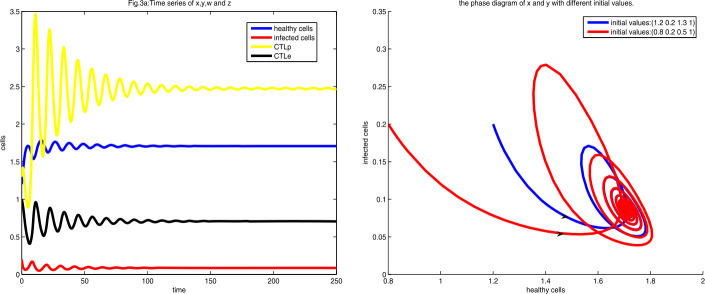
Figure 3(**a**) is the diagram of time series of *x*, *y*, *w*, and *z* at $a=1$, and the initial value is $(1.2, 0.2, 1.3, 1)$, whereas Fig. 3(**b**) is the phase diagram of *x* and *y* with different initial values

According to formulas (3.13), we obtain the Routh–Hurwitz determinants:

4.2 $$ \textstyle\begin{cases} \Delta _{2}(a)=\frac{171}{50}-\frac{58}{25}\sqrt{2}+\frac{3}{50}a, \\ \Delta _{3}(a)=\frac{3}{17\text{,}000}(-5+2\sqrt{2})(a-a_{-})(a-a_{+}), \end{cases} $$

where $a_{\pm }=\frac{3593}{34}\sqrt{2}-\frac{15\text{,}119}{102}\pm \frac{1}{102} \sqrt{461\text{,}988\text{,}655-326\text{,}669\text{,}598\sqrt{2}}$. Note that $\Delta _{2}(a)>0$ for all $a>0$ by (4.2), whereas if $a< a_{-}$, or $a>a_{+}$, then we have $\Delta _{3}(a)>0$. Thus $E_{2+}$ is locally stable by the Routh–Hurwitz theorem if $a_{-}< a<\frac{2+\sqrt{2}}{2}$. Let $a=1$. By simple calculation we obtain $E_{2+}=(1+\frac{1}{2}\sqrt{2}, \frac{3}{2}-\sqrt{2}, \frac{3}{10} \sqrt{2}(3+2\sqrt{2}), \frac{1}{2}\sqrt{2})$ and $\alpha _{1E_{2+}}\approx 0.8858$, $\alpha _{2E_{2}}\approx 0.5343$, $\alpha _{3E_{2}}\approx 0.2743$, $\alpha _{4E_{2}}\approx 0.0515$, $\Delta _{2E_{2}}\approx 0.1990$, $\Delta _{3E_{2}}\approx 0.0142$ by (4.1) and (4.2). Therefore it is locally stable, as shown in Fig. 3.

As can be seen in Fig. 3(a) and Fig. 3(b), each cell first shocks and then settles to equilibrium $E_{2+}$.

Now we verify the Hopf bifurcation by Theorem 3.4. The formulas given by (3.14) are

4.3 $$ \textstyle\begin{cases} A_{2}=3-\frac{1}{2}\sqrt{2}-a, \\ B_{2}=\frac{11}{2}-\frac{17}{4}\sqrt{2}+\frac{1}{2}a, \\ C_{2}=\frac{3}{2}-\sqrt{2}, \\ A_{3}=-\frac{87}{8}a+\frac{21}{2}+\frac{29}{4}\sqrt{2}-\frac{29}{4} \sqrt{2}a+\frac{11}{4}a^{2}+\frac{15}{8}\sqrt{2}a^{2}, \\ B_{3}=-\frac{1}{544}(15\sqrt{2}+22)[a-(-\frac{407}{34}+\frac{657}{68} \sqrt{2}-\frac{1}{68}\sqrt{1\text{,}566\text{,}150-1\text{,}107\text{,}132\sqrt{2}})] \\ \hphantom{B_{3}={}}{}\times [a-(-\frac{407}{34}+\frac{657}{68}\sqrt{2}+\frac{1}{68} \sqrt{1\text{,}566\text{,}150-1\text{,}107\text{,}132 \sqrt{2}})], \\ C_{3}=-\frac{1}{272}(-22+15\sqrt{2})(16\sqrt{2}+28-17a). \end{cases} $$

It follows that the existence conditions of positive equilibrium $E_{2+}$ ($a\in (0,\frac{1}{4})\cup (\frac{1}{4},\frac{2-\sqrt{2}}{2})$ or $a\in (\frac{2-\sqrt{2}}{2},\frac{2+\sqrt{2}}{2})$) also directly guarantee that $A_{3}>0$ and $C_{3}>0$ (Theorem 3.4 (2)).

Since $B_{3}<0$ (Theorem 3.4 (2)), we have

$$ a>-\frac{407}{34}+\frac{657}{68}\sqrt{2}+\frac{1}{68} \sqrt{1\text{,}566\text{,}150-1\text{,}107\text{,}132 \sqrt{2}}\approx 2.155 $$

or

$$ 0< a< -\frac{407}{34}+\frac{657}{68}\sqrt{2}-\frac{1}{68} \sqrt{1\text{,}566\text{,}150-1\text{,}107\text{,}132 \sqrt{2}}\approx 1.225. $$

Moreover, according to (3.14), we obtain

4.4 $$ \begin{aligned} \Delta _{4}&= \frac{1}{36\text{,}992}(467+330\sqrt{2}) (-20+17a-8\sqrt{2})^{2} \\ &\quad {}\times\biggl[a-\biggl(-135+ \frac{193}{2}\sqrt{2}+2\sqrt{9184-6494\sqrt{2}} \biggr)\biggr] \\ &\quad {}\times \biggl[a-\biggl(-135+\frac{193}{2}\sqrt{2}-2\sqrt{9184-6494\sqrt{2}}\biggr) \biggr], \end{aligned} $$

whereas $\Delta _{4}=0$ yields

$$ \begin{aligned} a&=-135+\frac{193}{2}\sqrt{2} \pm 2\sqrt{9184-6494\sqrt{2}}, \frac{8\sqrt{2}+20}{17}, \frac{8\sqrt{2}+20}{17} \\ &\approx 2.0949, 0.8483, 1.8420, 1.8420. \end{aligned} $$

Denote $a^{*}=-135+\frac{193}{2}\sqrt{2}-2\sqrt{9184-6494\sqrt{2}}\approx 0.8483$ ($\in (\frac{2-\sqrt{2}}{2},\frac{2+\sqrt{2}}{2})$). Intersecting with the existence condition of equilibrium $E_{2+}$ and $B_{3}<0$, we obtain $\Delta _{4}<0$ for $a\in (a^{*}, \frac{2+\sqrt{2}}{2})$ and $\Delta _{4}=0$ for $a=a^{*}$, whereas $\Delta _{4}>0$ for $a\in (0, \frac{1}{4})\cup (\frac{1}{4}, \frac{2-\sqrt{2}}{2})\cup ( \frac{2-\sqrt{2}}{2}, a^{*})$, which implies that the two roots of $\Delta _{3}=0$ are (by (3.13))

4.5 $$ h_{1}^{*}=\frac{-B_{3}-\sqrt{\Delta _{4}}}{2A_{3}},\qquad h_{2}^{*}= \frac{-B_{3}+\sqrt{\Delta _{4}}}{2A_{3}}, $$

where $A_{3}$ and $B_{3}$ are given in (4.3), and $\Delta _{4}$ is given in (4.4). By Theorem 3.3, $E_{2+}$ is stable if $h\in (0,h_{1}^{*})\cup (h_{2}^{*},+\infty )$. Submitting $a=0.5$ into (4.5), we get $h_{1}^{*}\approx 0.03872$ and $h_{2}^{*}\approx 0.2728$, and therefore $E_{2+}$ is stable if $h=0.3\ (\mbox{given number}) >h_{2}^{*}$. For $a=0.31$, we have $h_{1}^{*}\approx 0.029245$ and $h_{2}^{*}\approx 0.304727$, which implies that $h=0.3$ (given number) $\in (h_{1}^{*},h_{2}^{*})$, and thus $E_{2}$ is unstable.

**Figure 4:**
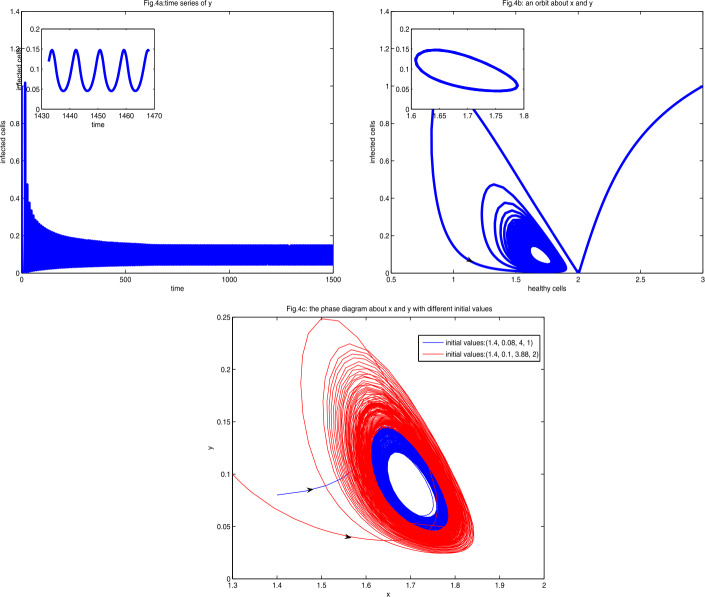
Figure 4(**a**) is the diagram of time series of *y* at $a=0.31$ with initial value $(3,1,2,4)$. Figure 4(**b**) is a part of the phase diagram about *x* and *y* with the same initial values. Figure 4(**c**) is the phase diagram with different initial values

Fix $a=0.31$ and take *h* as the bifurcation parameter. By Theorem 3.4 there are two Hopf bifurcations at $h=h_{1}^{*}\approx 0.029245$ and $h=h_{2}^{*}\approx 0.304727$, which implies that two limit cycles occur. With the help of Matcont [38], we obtain that the first Lyapunov coefficient is −0.07004781 as $h=h_{1}^{*}\approx 0.029245$, whereas another first Lyapunov coefficient is −0.001386494 as $h=h_{2}^{*}\approx 0.304727$, and thus the two Hopf bifurcations are supercritical, and the limit cycles are stable. The phrase diagrams of system are shown in Fig. 4.

As can be seen in Figs. 4(a), 4(b), and 4(c), a stable limit cycle appears, and stable periodic solutions bifurcate from it.

We must point out that when $a\in (0, \frac{1}{4})\cup (\frac{1}{4}, \frac{2-\sqrt{2}}{2})$, the infectious equilibrium $E_{1}$ without CTL is stable, whereas the infectious equilibrium $E_{2+}$ with CTL exists. To display this case, let $a=0.27$ and $h=0.4$, which indicates that both $E_{1}$ and $E_{2+}$ are stable. By direct calculation we obtain $E_{1}\approx (0.27, 3.2037, 0, 0)$ and $E_{2+}\approx ( 1.7071, 0.0858, 1.4371, 6.7009)$, and we draw the diagrams with different initial values.

**Figure 5:**
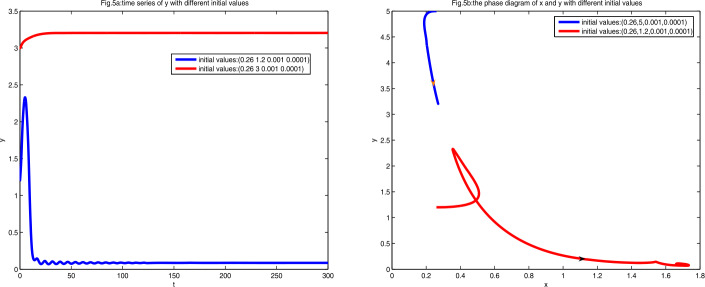
Figure 5(**a**) is the diagram of time series of *y* at $a=0.27$ and $h=0.4$ with different initial values. Figure 5(**b**) is the phase diagram of *x* and *y* with different initial values

As shown in Figs. 5(a) and 5(b), there are two stable equilibria $E_{1}$ and $E_{2+}$, that is, bistability occurs, and the infected cells converge to one of them depending on the initial values.

Besides, when equilibrium $E_{2+}$ is unstable, then a stable limit cycle occurs, and $E_{1}$ is still stable. To display this phenomenon directly, letting $a=0.1$ and $h=0.3$, we draw the diagrams with different initial values.

**Figure 6:**
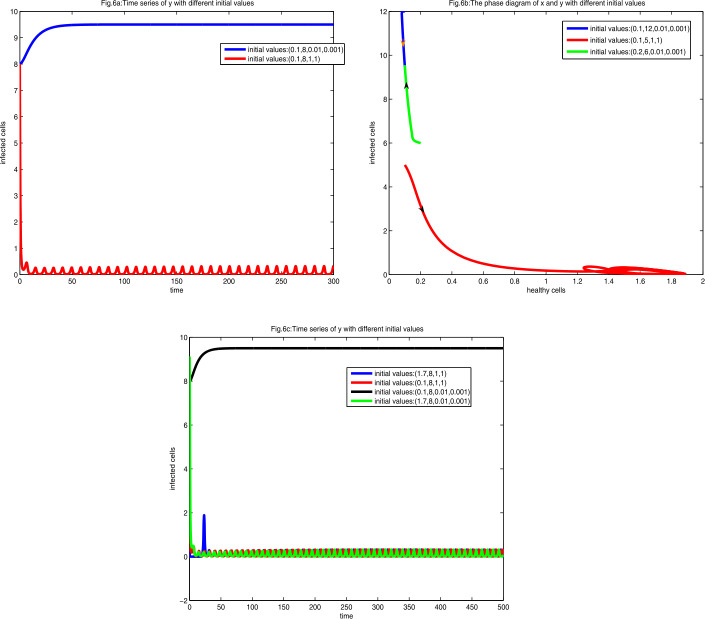
Figure 6(**a**) is the diagram of time series of *y* at $a=0.1$ and $h=0.3$ with different initial values. Figure 6(**b**) is the phase diagram of *x* and *y* with different initial values. Figure 6(**c**) is the diagram of time series of *y* with different initial values

As shown in Figs. 6(a) and 6(b), a stable equilibrium $E_{1}$, an unstable equilibrium $E_{2+}$, and a stable limit cycle, which is bifurcated from $E_{2+}$, appear, and the infected cells converge to one of them depending on the initial values.

For the case of $C_{3}<0$, in Theorems 3.3 and 3.4, we give another set of parameters $d=3.6=b$, $q=1$, $c=28.8$, $h=0.8$. We do not discuss it now.

## Discussion

This paper studies an HIV model proposed by Wodarz et al. [31, 32] to describe the interaction between healthy cells and infected cells as well as primary and secondary immune response. Compared with [33], in this model, we assume that the production of primary immune response is not only connected with infected cells but also with healthy cells. We also assume that virus at its steady state is proportional to infected cells. The structure of equilibria is analyzed in [31, 32]. But for a higher-dimensional system, stability and bifurcation analysis is important and complex for the full range of possibilities. Because of adding the healthy cells to the produced CTLp, the model shows rich dynamic behavior on stability and bifurcations.

It is interesting that this model displays the bistability phenomenon, that is, two stable equilibria $E_{1}$ and $E_{2+}$ or a stable equilibrium $E_{1}$ and a stable limit cycle, which is bifurcated from the unstable equilibrium $E_{2+}$. Which one is stable not only depends on relationship of parameters but also depends on the initial values of cells. As shown in Figs. 5(a) and 5(b), high initial virus load close to $E_{1}$ leads to the convergence to $E_{1}$, which means that CTL memory fails to establish. Low initial virus load close to $E_{2+}$ leads to the convergence to $E_{2+}$, CTL memory successfully establishes, and the virus load first increases and then decreases to stay at a low level. Therefore we must increase dosage to inhibit the virus replication in a brief period and help the immune response establish. As shown in Fig. 6(a), if the initial healthy cells and virus load are the same, and high initial CTL account means that the CTL response establishes, virus load may decrease at primary process, which was considered as the CTL clear away some virus, but over time, it oscillates and cannot be completely eradicated. This phenomenon can be viewed as an individual having a chronic disease that may flare up from time to time, and it is a long struggle between virus and immune response. Initial values with little CTL lead to high virus load. As can been in Fig. 6(c), high or low initial healthy cells do not change the development of disease. However, high initial healthy cells first lead to decreasing the virus load to a very low state, which is almost clear away the virus, which means that the healthy cells play an important role in clearing away the virus under certain circumstances.

The interaction of virus and the host immune system is a complicated and long process as HIV has a long latent period, and the disease cannot cure completely. We have shown rich dynamic patterns, but the model considered here is just a simple one. It is easy to improve and expand the model. For example, we can add the virus equation in model (1.3) (see a more detailed description in [32]), we may consider delay in this model as in [39], drug treatment [13], or latent cells [40] mentioned in the introduction. Such modifications should more precisely react the reality and give us more advice in understanding the infection process, which leads to a more challenging mathematical analysis.
